# Association between response to triptans and response to erenumab: real-life data

**DOI:** 10.1186/s10194-020-01213-3

**Published:** 2021-01-06

**Authors:** Ilaria Frattale, Valeria Caponnetto, Alfonsina Casalena, Maurizio Assetta, Maurizio Maddestra, Fabio Marzoli, Giannapia Affaitati, Maria Adele Giamberardino, Stefano Viola, Amleto Gabriele, Francesca Pistoia, Davide Cerone, Carmine Marini, Simona Sacco, Raffaele Ornello

**Affiliations:** 1 Neuroscience Section, Department of Applied Clinical Sciences and Biotechnology, University of L’Aquila, Via Vetoio 1, 67100 L’Aquila, Italy; 2 Department of Neurology, ‘G. Mazzini’ Hospital, Teramo, Italy; 3 Department of Neurology, ‘F. Renzetti’ Hospital, Lanciano, Italy; 4 Department of Medicine and Science of Aging, ‘G. D’Annunzio’ University, Chieti, Italy; 5 Department of Neurology, ‘S. Pio da Pietrelcina’ Hospital, Vasto, Italy; 6 Neurology Service, ‘SS.Annunziata’ Hospital, Sulmona, Italy; 7 Department of Neurology, ‘S. Salvatore’ Hospital, L’Aquila, Italy; 8 Department of Internal Medicine, Public Health, Life and Environmental Sciences, University of L’Aquila, L’Aquila, Italy

**Keywords:** Triptans, Erenumab, Migraine treatment, CGRP

## Abstract

**Background:**

Triptans and erenumab are both migraine-specific agents acting on the calcitonin gene-related peptide pathway. Therefore, response to triptans might be associated with response to erenumab.

**Main body:**

In our study, consecutive patients referring to the Headache Centers of the Abruzzo region from January 2019 to March 2020 and treated with erenumab were interviewed about past use and efficacy of triptans. Triptan users were classified as ‘triptan responders’ if they were headache-free 2 h after treating ≥3 migraine attacks with ≥1 triptan. We considered patients as ‘erenumab responders’, if they had a ≥ 50% mean reduction in monthly migraine days between the 4th and the 6th month from treatment start compared with baseline. Of 91 triptan users, 73 (80.2%) were triptan responders and 58 (63.7%) were erenumab responders. The odds ratio of being erenumab responder was 3.64 (95% CI, 1.25–10.64) for triptan users as compared to non-users. (*P* = 0.014). Besides, starting erenumab improved triptan response in both erenumab responders and non-responders.

**Conclusions:**

Our data of an association between response to triptans and response to erenumab can be useful for patient advice and to improve the understanding of migraine pathophysiology and treatment.

## Background

Migraine affects 14.4% of adults worldwide [1]. Despite the high burden of migraine, preventive treatments were not disease-specific until the advent of monoclonal antibodies targeting the calcitonin gene-related peptide (CGRP) pathway [2, 3]. During a migraine attack, the activation of the trigeminovascular system has a key role, leading to CGRP release at the trigeminal endings; this leads in turn to vasodilation of the intracranial arteries, modulates neuronal excitability through the facilitation of pain transmission, and activates neurogenic inflammation [3, 4]. Monoclonal antibodies targeting CGRP or its receptor lead to modulation of pain transmission and reduction in both peripheral and central sensitization by removing the excess of released CGRP (anti-CGRP antibodies) or the block of the ligand from binding the CGRP receptor (anti-CGRP receptor antibodies) [4].

While the development of migraine-specific preventive treatments is recent, acute treatments that are specific for migraine are available since the 1990s. Triptans are agonists of the 5-hydroxytryptamine (5-HT) receptors 5-HT1B, 5-HT1D and 5-HT1F [5]. 5-HT1B and 5-HT1D receptors are localized in trigeminal ganglia and trigeminal nerves; 5-HT1D receptor are detected in trigeminal nerves projecting peripherally to the dural vasculature to inhibit activated trigeminal nerves and prevent vasoactive neuropeptide release, and centrally to the brainstrem trigeminal nuclei to interrupt pain signal transmission [6]. At the peripheral level, triptans constrict extracerebral blood vessels and reduce trigeminal sensory nerve activation, thus ultimately inhibiting vasoactive peptide release including substance P and CGRP [7]. Therefore, triptans indirectly share their target with monoclonal antibodies targeting the CGRP pathway [8]. Hence, it can be speculated that migraineurs whose attacks respond to triptans might also have a favorable response to the new migraine-specific preventive treatments.

In the present real-life study, we assessed 1) whether previous response to triptans predicts the subsequent efficacy of erenumab; 2) whether the loss of efficacy – wear-off – of triptans over time predicts erenumab ineffectiveness, and 3) whether erenumab treatment improves the efficacy of triptans.

## Methods

This is an ancillary study from a real-life observational study on patients treated with erenumab [9]. The study was approved by the Internal Review Board of the University of L’Aquila with protocol number 44/2019. The study database is available from the Corresponding Author upon reasonable request.

Our study included patients aged 18 to 65 years and consecutively treated with erenumab from January 2019 to March 2020 in the Headache Centers of Avezzano, L’Aquila, Sulmona, Teramo, Chieti, Lanciano, and Vasto. All patients had a diagnosis of migraine with or without aura according with International Classification of Headache Disorders (ICHD) criteria [10]. According to clinical indications for erenumab, we included patients with chronic or high-frequency (≥8 monthly headache days) episodic migraine. For each patient we recorded sex, age, and migraine characteristics.

We collected information on the use of triptans by means of a structured data collection form administered via telephone interview. The data collection form was designed by consensus among the study participants according to their clinical experience. Patients were asked about past use of the six triptans available in Italy, namely almotriptan, eletriptan, frovatriptan, rizatriptan, sumatriptan, and zolmitriptan, at any time before starting erenumab treatment. Information was checked with medical chart review where available. We only included patients who signed a written informed consent to participate in an observational study on monoclonal antibodies targeting the CGRP pathway; oral additional consent prior to the questionnaire was deemed sufficient to participate in the present study. Patients were considered not available on the phone if not answering after three phone call attempts.

Patients who reported use of at least one triptan for at least three migraine attacks were considered triptan users. Triptan users were classified as ‘triptan responders’ if they were headache-free within 2 h after treating at least three migraine attacks with one triptan [11]; the remaining patients were classified as ‘triptan non-responders’. Triptan responders were also asked about a decreased response to triptans over time, configuring the ‘wear-off’ phenomenon. Those same patients were also asked whether the efficacy of triptans improved after starting treatment with erenumab.

Regarding erenumab treatment, patients were classified as ‘erenumab responders’ if reporting a mean ≥ 50% reduction in monthly migraine days from baseline (i.e. the three months preceding the start of erenumab treatment) to month 4–6 of treatment, in accordance with a secondary end-point of the STRIVE trial [12]; the remaining patients were classified as ‘erenumab non-responders’.

### Statistical analysis

We included in primary analyses all triptan users. We reported descriptive statistics by using numbers and proportions or means and standard deviations (SDs) as appropriate. We reported the odds ratios (ORs) and 95% confidence intervals (CIs) of the association between triptan response and erenumab response by means of the chi squared statistics. According to the calculations made with G*Power software [13], we estimated that performing a chi squared analysis with a total sample size of *n* = 88 would be sufficient to detect a medium (f = 0.3) effect size between two groups with a *P* value < 0.05 and 80% power. We planned subgroup analyses on 1) patients using triptans during the three months before starting erenumab treatment and 2) patients who continued using triptans during erenumab treatment. We used Microsoft Excel and SPSS version 20 to perform the analyses.

## Results

**Fig. 1:**
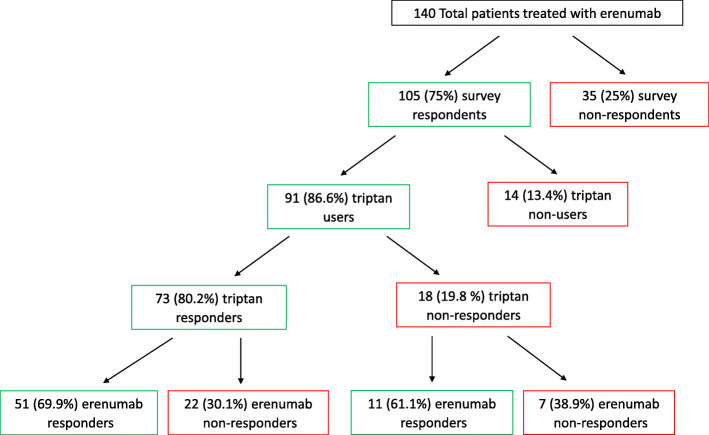
Flowchart of patient inclusion

**Fig. 2:**
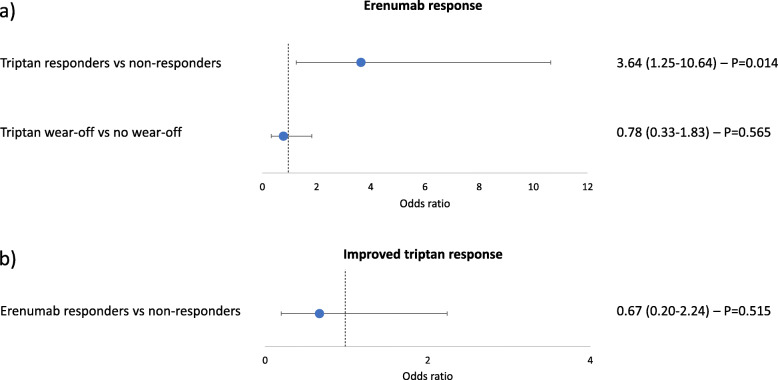
**a** Odds ratios and 95% confidence intervals of erenumab response according to triptan response and triptan wear-off. **b** Odds ratios and 95% confidence intervals of improvement in triptan response according to erenumab response

During the study period, 140 patients were treated with erenumab for at least 6 months; 105 of them (75.0%) answered to the data collection form, while 35 (25.0%) were not available on the phone. Among the 35 patients not answering to the data collection form, 16 (45.7%) were erenumab responders and 19 (54.3%) erenumab non-responders. Ninety-one (86.6%) of the 105 patients responding to the data collection form were considered triptan users and included in the study (Fig. 1). Regarding erenumab, 58 (63.7%) of the included patients were erenumab responders and 33 (32.3%) were erenumab non responders; regarding triptans, 73 (80.2%) were triptan responders and 18 (19.8%) triptan non-responders. There were no major differences in baseline characteristics between erenumab responders and non-responders (Table 1); the only significant difference regarded triptan use. In fact, among erenumab responders, 51 (87.9%) were triptan responders while among erenumab non-responders, 22 (66.7%) were triptan responders. The OR of being erenumab responder was 3.64 (95% CI, 1.25–10.64) for triptan responders as compared to non-responders (*P* = 0.014; Fig. 2-a).

**Table 1:** Comparisons between erenumab responders and non-responders in the 91 triptan users

	Erenumab responders (*n* = 58)	Erenumab non-responders (*n* = 33)	*P* value
*N, %*			
Female	49 (84.5)	30 (90.9)	0.384
Chronic Migraine	53 (91.4)	29 (87.9)	0.591
Medication overuse	38 (65.5)	23 (69.7)	0.683
Aura	21 (36.2)	10 (30.3)	0.568
Allodynia	33 (56.9)	15 (45.5)	0.293
Preventive treatment failures			0.464
2–4	36 (62.1)	23 (69.7)	
> 4	22 (37.9)	10 (30.3)	
Triptan responders	51 (87.9)	22 (66.7)	0.014
*Mean ± SD*			
Age	46.6 ± 9.5	46.6 ± 10.9	0.926
Migraine duration, years	25.0 ± 11.2	29.3 ± 12.3	0.143
Monthly headache days	21.8 ± 7.9	18.3 ± 9.4	0.084
Monthly migraine days	18.2 ± 7.1	17.8 ± 9.3	0.827
Monthly medication days	18.2 ± 8.7	18.6 ± 8.2	0.833
Mean headache intensity	7.8 ± 1.8	7.7 ± 1.7	0.792

Forty-six triptan users (50.5%) reported triptan wear-off. Among erenumab responders, 28 (48.3%) had triptan wear-off, while among erenumab non-responders 18 (54.5%) had triptan wear-off. The OR of being erenumab responder was 0.78 (95% CI, 0.33–1.83) for patients reporting triptan wear-off as compared to those not reporting triptan wear-off (*P* = 0.565; Fig. 2-a).

Sixty-five patients (71.4%) were triptan users during the three months preceding erenumab treatment; 60 (92.3%) of them were triptan responders, while the remaining 5 (7.7%) were triptan non-responders. Due to low numbers, we did not perform a subgroup analysis on those patients.

After starting erenumab treatment, 52 patients (57.1%) continued using triptans; 36 of them (69.2%) were erenumab responders and 16 (30.8%) erenumab non-responders. Twenty-nine (55.8%) patients in the overall group, 19 (52.8%) erenumab responders and 10 (62.5%) erenumab non-responders reported an improvement in triptan effectiveness. The proportion of patients reporting an improvement in triptan effectiveness was similar in erenumab responders and erenumab non-responders (52.8% vs 62.5%; OR 0.67; 95% CI, 0.20–2.24; *P* = 0.265; Fig. 2-b).

## Discussion

In our study, patients showing a favorable response at any time to at least one triptan had a higher probability to be responders to erenumab compared with those not responding to triptans. This information is important as it may improve our understanding of migraine pathophysiology and treatment; it could also be used in clinical practice to advise patients about their chances of response to erenumab treatment. However, previous response to triptans alone should not represent a strict criterion to select patients for erenumab treatment, because many triptan non-responders were erenumab responders.

To our knowledge, this is the first study primarily addressing the association between the response to triptans and that to monoclonal antibodies targeting the CGRP pathway. A previous real-life study found a trend toward better response to erenumab in triptan responders compared with non-responders [14], being however underpowered to draw definite conclusions. The remaining available real-life studies on the safety and efficacy of erenumab [15–20] did not assess triptan response. Notably, the proportion of patients responding to erenumab in our cohort was higher than in randomized clinical trials [12, 21], while it was comparable to other real-life studies [14–16]. Our proportion of triptan users was also higher than those of trials [12, 21], while no comparable data are available for real-life studies.

The findings of our study can be explained considering what we know about migraine pathophysiology. A common action on the trigeminovascular system [8] might explain the association between response to triptans and response to erenumab. A previous study found that patients responding to rizatriptan had higher jugular blood levels of CGRP during migraine episodes compared with patients not responding to rizatriptan; besides, patients responding to rizatriptan had a steep decrease in CGRP after the administration of rizatriptan, which was not found in non-responders [11]. In triptan non-responders, pain neurotransmitters different from CGRP might be important in the generation of migraine; thus, triptan non-responders might be less responsive to CGRP-targeted treatments. It is important to note that while erenumab was designed as a CGRP receptor blocker, the action of triptans on CGRP is indirect.

Our finding of an association between response to triptans and response to a migraine-specific preventative is in line with a previous report which found an association between response to triptans and response to onabotulinumtoxin A [22]. However, the association found by this early study was not confirmed in a further report [23]. The rationale for those studies was that onabotulinumtoxin A acts on the trigeminovascular system as well as triptans [8]. Onabotulinumtoxin A acts on peripheral nerves terminal to interfere with specific events in the synaptic vesicle cycle. These vesicles contain small molecules like acetylcholine and glutamate or vasodilatory neuropeptides including pituitary adenylate cyclase activating peptide 38 (PACAP 38), Substance P, and CGRP. Thus, onabotulinumtoxin A injection inhibits the release of these neuropeptides from primary sensory first order neurons in dorsal root and trigeminal ganglia [24]. During a migraine attack, the headache phase depends on activity in unmyelinated C- and thinly myelinated Aδ-fibers in the dura. Onabotulinumtoxin A appears to selectively inhibit the activation and sensitization of the unmyelinated C but not thinly myelinated Aδ fibers [25]. Preclinical evidence suggests that monoclonal antibodies acting on CGRP inhibits Aδ but not C type neurons in the trigeminal ganglion [26], therefore having an activity complementary to that of onabotulintoxinA. Taking all the evidence together, monoclonal antibodies acting on the CGRP pathway, triptans, and onabotulinumtoxin A all are involved in inhibition of the release of CGRP, but at the same time they also act on different pathways implicated in the pathogenesis of migraine.

Data about the improvement in response to acute medication are not reported by randomized clinical trials, despite being relevant in clinical practice as an additional efficacy outcome of migraine preventive medication. Improving responsiveness to acute medication is a goal of migraine prevention [27] and might be an additional parameter to test the efficacy of preventative drugs. In our study, after starting erenumab, more than half of patients reported an improvement in their response to triptans. This favorable effect was appreciated not only in erenumab responders but also in erenumab non-responders; on the contrary, among patients treated with onabotulinumtoxin A only responders reported an improved response to triptans [23]. The improved response to triptans found in patients treated with erenumab can be explained by synergy, as erenumab blocks the CGRP receptor, while the target of triptans is not the CGRP receptor itself. We cannot exclude that the improved response to triptans might also be explained by an overall improved response to acute medication, including triptans and non-steroidal anti-inflammatory drugs, in patients treated with a migraine preventative.

Our data are preliminary and should be taken with caution, as they come from a real-life, non-randomized study. Our study is also limited by a small sample size, allowing reliable univariate comparisons but not multivariate adjustments. The limited number of patients treated with triptans during the three months before erenumab treatment and after starting the treatment also limited the possibility of performing subgroup analyses. Despite the high rate of response to the questionnaire (75%), ensuring the reliability of our sample, the retrospective recall of information through telephone interview is prone to recall bias. Moreover, due to the limited number of patients we could not address differences according to the different triptans. Lastly, our data only refer to patients treated with erenumab, a CGRP receptor antagonist, and are therefore not generalizable to monoclonal antibodies targeting the CGRP molecule.

## Conclusion

According to our real-life data, patients reporting response to at least one triptan have a higher likelihood to respond to erenumab treatment compared with triptan non-responders. This information is relevant to improve our understanding of migraine and its treatments and to predict the efficacy of migraine-specific preventatives.

## Data Availability

anonymized data operated or analyzed during this study are available from the Authors upon reasonable request.

## Abbreviations

5-HT: 5-hydroxytriptamine
CGRP: Calcitonin gene-related peptide
ICHD: International Classification of Headache Disorders
STRIVE: Study to Evaluate the Efficacy and Safety of Erenumab (AMG 334) in Migraine Prevention
